# Risk Factors Associated with Outcome of Spontaneous Intracerebral Haemorrhage: Hospital Kuala Lumpur Experience

**DOI:** 10.21315/mjms2022.29.1.8

**Published:** 2022-02-23

**Authors:** Rajendra Rao Ramalu, Regunath Kandasamy, Azman Raffiq, Zamzuri Idris, Johari Siregar Adnan

**Affiliations:** 1 Department of Neurosciences, School of Medical Sciences, Universiti Sains Malaysia, Kelantan, Malaysia; 2 Department of Neurosurgery, Hospital Tuanku Ampuan Afzan, Pahang, Malaysia; 3 Department of Neurosurgery, Penang General Hospital, Pulau Pinang, Malaysia; 4 Department of Neurosurgery, Hospital Sungai Buloh, Selangor, Malaysia

**Keywords:** haemorrhagic stroke, spontaneous intracerebral haemorrhage, clinical profile, outcome, Malaysia

## Abstract

**Background:**

Stroke is the third leading cause of death in Malaysia. This study aims to evaluate the mortality risk factors of spontaneous intracerebral haemorrhage (ICH) in Hospital Kuala Lumpur, Malaysia.

**Methods:**

A single centre prospective cohort study was performed between 1 May 2017 and 30 April 2018 for patients above 18 years old with spontaneous ICH. Patients exhibiting haemorrhage due to tumours and ruptured arteriovenous malformations were excluded. The demographics, clinical parameters, radiology findings, surgical intervention, mortality at 30 days and outcome based on modified Rankin score (MRS) grading were recorded. Selected variables were incorporated into a binary logistic regression to determine the outcome predictors of mortality.

**Results:**

A total of 292 patients were recruited into the study. The findings showed that the mean age of ICH patients was 48.9 years old. Most of the lesions were located in the putamen (45.9%). More than half (61.0%) of the patients presented with a good outcome whereas 39.0% of the patients presented with a poor outcome. The mortality rate at 30 days was 29.1%. The important predictors for mortality were male (odds ratio [OR] = 0.343; *P* = 0.043), concomitant use of warfarin and aspirin (OR = 14.696; *P* = 0.007), ischaemic heart disease [IHD] (OR = 0.007; *P* = 0.003), brainstem bleed (OR = 0.001; *P* = 0.001), the presence of intraventricular haemorrhage [IVH] (OR = 0.198; *P* = 0.049) and surgery.

**Conclusion:**

Most diagnosed ICH patients in Malaysia were at a younger age (mean of 48.9 years old) with the significant mortality predictors were IVH, IHD, surgery and brainstem bleed.

## Introduction

Haemorrhagic stroke or intracerebral haemorrhage (ICH) is an important cause of death and disability worldwide. ICH also contributed to higher morbidity compared to cerebral infarction or subarachnoid haemorrhage (1). The Malaysian Acute Stroke Registry reported a 25.9% of 1-month mortality among patients presented with ICH (2).

A study has shown that Caucasians in the Asian population has a 5 times higher incidence of developing ICH compared to the Australian population (3). This finding was confirmed by the Malaysia stroke registry documentation whereby the mean age for ICH was 60.8 years old, which is 10 years younger than the developed countries (2).

The ICH score is a simple clinical grading scale that allows ICH risk stratification to predict mortality at 30 days (1). However, certain factors included in the ICH score are vague and broad.

Firstly, a cumulative Glasglow coma scale (GCS) is used instead of a specific scoring system. In the cumulative GCS, there is an absence of exclusion criteria for the patient that has an ICH over the Broca’s area (that would result in aphasia) hence skewing the result to an unfavourable prognosis.

Secondly, no concomitant co-morbid was considered. Based on our clinical experience, ICH is more common in patients with renal disease and cardiac problems (atrial fibrillation patients on anti-platelet therapy or warfarin). These patients are also common in the poor group category compared to those without these co-morbid.

Through observation, younger patients presenting with ICH and patients with multiple co-morbid had a poor outcome. Therefore, this study aims to investigate and identify the risk factors involved in ICH. In the Malaysian context, no recent study was performed over the past 5 years regarding ICH.

The most relevant recent data was retrieved from the Malaysian Acute Stroke Registry Malaysia in which the data was collected from 2010 to 2014. However, concomitant co-morbid such as chronic kidney disease or valvular heart disease was not included. Moreover, the data on the patient undergoing surgical intervention and the outcome of the intervention was missing (2).

This study aims to determine and evaluate the associated risk factors for mortality of ICH in Hospital Kuala Lumpur. This will aid us in our clinical practice for a better clinical decision.

## Methods

### Research Design

The main objective is to analyse the associated risk factors of mortality of spontaneous ICH at 30 days.

### Research Location and Duration

This single-centre study was conducted in Hospital Kuala Lumpur. Data was collected from 1 May 2017 until 30 April 2018.

### Study Population

All patients with spontaneous ICH aged 18 years old and above referred to the neurosurgery Hospital Kuala Lumpur were included in this study. The diagnosis was based on a typical clinical onset and computed tomography (CT) brain. ICH definitions and rules were specified by the Tenth Revision of the International Classification of Diseases (ICD-10) under code I61.0 for non-traumatic intracerebral haemorrhage, unspecified were applied.

Patients with secondary intracerebral haemorrhage (due to vascular or brain tumour aetiology) were ruled out. Any patient less than 45 years old with no risks for hypertensive haemorrhage was investigated with a CT angiogram (CTA) after 6 weeks (4). Patients with positive CTA findings were excluded from the study. Patients that fulfilled the inclusion and exclusion criteria were enrolled in this study.

### Research Method

The parameters of interest in this study were age, gender, race and co-morbid such as: i) hypertension: previous usage of antihypertensive drugs or previous medical diagnosis of hypertension; ii) diabetes mellitus: fasting glucose value of 6.7 mmol/L at the time of admission or corresponding value for random plasma glucose of 17.8 mmol/L at the time of admission; iii) ischaemic heart disease or history of cerebrovascular disease; iv) chronic kidney disease (based on Cockgroft Gault formula with stage 4 renal failure or worst); v) valvular heart disease (if any valve surgery confirmed with surgery performed) and clinical parameters such as blood pressure (BP) and GCS upon admission, radiological imaging (site, location of ICH, presence of IVH and hydrocephalus), surgical intervention performed (craniotomy, craniectomy or extraventricular drainage [EVD]) and finally the outcomes of mortality or morbidity at 30 days.

The multivariate logistic regression analysis of this study consists of these 19 independent variables. It has been suggested that in the concept of minimum 10 number of events per variable (EPV) is needed in multivariate logistic regression analysis to avoid bias and error (5, 6). Therefore, sample size population estimation with 19 variables is calculated as below:

The minimum total sample size required:


19 variables×10 event per variable=190 samples

Re-calculate drop-out rate based on this formula with an estimation drop-out rate of 20%

Final sample size


$$\begin{array}{l} =\frac{100\%\;\text{minimum samples size}}{100\%-\text{drop out}\%}\\ =\frac{190}{1-0.2}\\ =238 \end{array}$$

Note: The sample size calculated above is not the upper limit for the sample size, ideally it refers to the minimum number required in the analysis of multivariate logistic regression. This calculation is performed according to the number of variables to avoid bias and error.

The classification of each haematoma location was based on the haematoma location of the epicentre whether it is supratentorial (lobar, thalamic and putaminal), infratentorial (cerebellar or brain stem) or others (midline or bilateral). The associated condition on the CT scan such as intraventricular bleed and hydrocephalus was documented. Any supratentorial ICH (basal ganglionic and thalamic haemorrhage) condition was scored further using Kuroda and Kanaya (7) classification. The intracerebral haematoma volume was estimated by measuring the greatest diameter ‘A’ and perpendicular diameter ‘B’ of haematoma, and the thickness of each CT slice by adding the number of CT slices visualising haematoma. These values are multiplied and the product (A × B × C) is divided by two to yield the approximated volume of clot based on ABC/2 (8).

Management of ICH was in accordance with a previous protocol (9). Medical therapy was performed with BP stabilisation as per American Heart Association (AHA) guidelines and referral to rehabilitation team if motor weakness is present. Patients with mild symptoms and who refused surgical intervention were treated conservatively. Patients with hydrocephalus or intraventricular haemorrhage according to CT imaging were subjected to ventricular drainage (10). Evacuation of clot was offered to patient with obvious hemiplegia and altered consciousness (based on GCS score). However, the clot size must be more than 30 cc within 1 cm from the cortical surface (7). The decision to operate was not affected by the position of the haemorrhage (dominant or non-dominant hemisphere) or by the presence or absence of aphasia. There was no absolute upper age limit for the surgery. The outcome was assessed by a follow-up at 1 month using modified Rankin score (MRS). Discharged patient or their family members will be contacted to carry out a simplified modified Rankin scale questionnaire (sMRSq) scoring (8). The clinical outcome was divided into good outcome in which the patients made a good recovery (correspond to MRS score of 1–3) or poor outcome (based on MRS score of 4–6).

### Statistical Analysis

Statistical analysis was performed using IBM SPSS Statistics version 20.0. Numerical variables will be presented in mean and standard deviation whereas categorical variables will be presented in frequency and percentage. Univariate analysis was performed using simple logistic regression for both morbidity and mortality. Differences with a *P*-value of less than 0.05 were considered statistically significant. Significant data were included in binary logistic regression analysis and poor risk factors were identified for mortality. Assumptions for binary logistic regression are as follow (11):-

- The outcome is a binary or dichotomous variable such as yes versus no, positive versus negative, 1 versus 0.
- There is a linear relationship between the logit of the outcome and each predictor variable.
- There are no influential values (extreme values or outliers) in the continuous predictors.
- There are no high intercorrelations (such as multicollinearity) among the predictors.

The results were presented in a table including the adjusted odds ratio (OR) with 95% confidence interval (CI) and the corresponding *P*-value.

## Results

### Demographic

A total of 300 patients were recruited in this study from 1 May 2017 to 30 April 2018. arteriovenous malformation (AVM) was detected in eight patients (2.7%) through brain CTA 6 weeks resulting in a final total of 292 patients were enrolled in this study.

**Figure 1:**
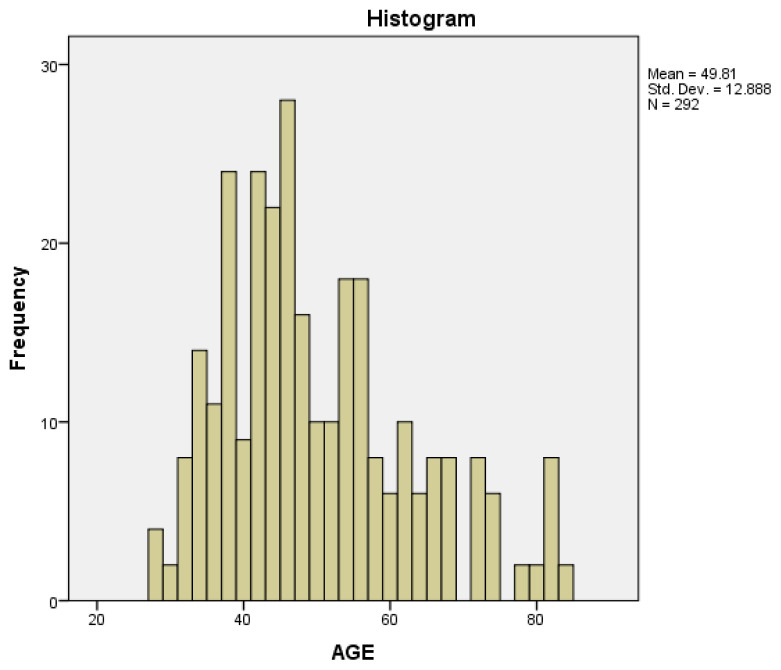
Age distribution noting most incidence of ICH occurring between 31 years old and 60 years old. Mean = 49.81 with median = 46.50 thus there is a slight skew to the right (positive)

The mean age of ICH was 49.8 years old with a median age of 46.5 years old. The age distribution histogram shows that the highest incidence of ICH occurs between the age of 31 years old and 60 years old. The higher value of mean compared to median proves the skewness of the data to the right (positive) suggesting younger age with ICH association in this study (Figure 1).

There were 182 males (62.3%) and 110 females (37.7%) in this study with a ratio of 1.65:1. There were 215 Malay patients (73.6%), 54 Chinese patients (18.5%), 20 Indian patients (6.8%) and 3 other patients (1.1%). The age, patient’s gender and ethnicity did not show any statistical significance for mortality or poor outcome (Table 1). However, male (*P* = 0.063) and Indian race (*P* = 0.055) showed a nearly statistically significant for mortality.

### Clinical Profile

Multiple associated risk factors have been identified with ICH with the most common risk factor was hypertension (99.0%). The mean systolic blood pressure (SBP) and diastolic blood pressure (DBP) on admission were 207.4 mmHg and 105.8 mmHg, respectively. However, blood pressure showed non-statistically significant for mortality or poor MRS outcome.

Patient that consumed both aspirin and warfarin had a significant risk for mortality (*P* = 0.031) compared to the patient who consumed single drug.

In addition, the presence of ischaemic heart disease (IHD) and chronic kidney disease (CKD) were significant predictors for poor outcome and mortality. Meanwhile, valvular heart disease was significant for poor MRS outcome but not mortality (Table 1). The presence of diabetes also showed non-statistically significant.

Based on the symptoms, 193 patients (66.1%) presented with a history of loss of consciousness, 167 patients (57.2%) had a headache, 143 patients (49.0%) exhibited muscle weakness, 147 patients (50.3%) experiencing vomiting, 125 patients (42.8%) had slurred speech and 2 patients (0.7%) had a seizure. However, none of the symptoms was significant to predict poor outcome or mortality.

The mean GCS upon admission was 11.53. The GCS score recorded was 67 patients (22.9%) had a severe score, 97 patients (33.2%) had a moderate score, 69 patients (23.6%) had a mild score, 59 patients (20.2%) had a normal score of 15. Unfortunately, 53 patients that had severe GCS scores passed away. Severe GCS score upon admission had a strong statistical association with mortality and poor outcome (*P*-value = 0.0001) compared to the patient with a full GCS score.

Moderate GCS score showed a statistically significant in poor outcome (*P* = 0.003) whereby 33 patients admitted with moderate GCS score had a poor outcome. Among these 33 patients, 19 patients passed away (Table 1).

### Radiological Imaging

Most lesions were located in the basal ganglia (45.9%) followed by brainstem (18.2%), thalamic region (15.4%), cerebellar (10.6%) and lobar (9.9%). Patient with brainstem bleed had a very poor outcome with only 4 still alive among the 53 patients, but with poor MRS scores. Although thalamic bleed was not a significant predictor for mortality, it was significant for a poor outcome. Only 21 out of the 45 patients with thalamic bleed presented with a good MRS score.

Based on the location of the lesion, 125 patients (42.8%) had ICH lesions on the right side, 86 patients (29.5%) had ICH lesions on the left while the remaining 81 patients (27.7%) had ICH lesions over the midbrain, midline or both sides. Due to the strong predisposition of brainstem bleed for mortality, this has contributed to a statistically significant parameter of bleed site.

Around 17 patients (5.8%) had a midline shift (MLS) of more than 5 mm while 275 patients (94.2%) had MLS less than 5 mm. Although the presence of MLS was not a significant predictor for mortality, it was significant for poor outcomes. Only 10 patients from the 17 patients with MLS more than 5 mm survived. However, only four patients had a good MRS outcome.

Among the patients with ICH, 142 patients (48.6%) had a clot size less than 15 cc, 111 patients (38.0%) had a clot size between 15 cc and 30 cc, 29 patients had a clot size between 30 cc and 50 cc and 10 patients (3.4%) had a clot size more than 50 cc. Patient with a clot size of more than 50 cc showed a significant outcome for mortality and poor outcome. Among the 10 patients who presented with clots more than 50 cc, only 6 patients survived while only 4 patients presented with a good MRS score.

A total of 149 patients (51%) had intraventricular haemorrhage through CT scan observation. The presence of IVH was not statistically significant for mortality or poor outcome.

Furthermore, 55 patients (18.8%) had hydrocephalus (HCP) based on the CT scan and of these patients, 25 patients passed away and it showed a statistically significant (*P* = 0.004). Among the remaining 30 patients who survived, only 24 patients had a good MRS score.

The location of ICH in the brainstem, clot size of more than 50 cc and the presence of hydrocephalus showed a statistical significance for mortality and poor outcome. Meanwhile, the presence of thalamic bleed was significant for poor MRS outcome but was not significant for mortality (Table 2). Although the side of the bleed (others) was statistically significant, this could be a false positive due to the subset of the patient with brainstem bleed, which was categorised here.

A further category of radiology classification was done based on the study performed by Kuroda and Kanaya (7) in which 25.2% was in grade V followed by grade IIIb (23.5%), IVb (21.8%), II (10.1%), IIIa (7.3%) and, I and IVa at 6.1% of all supratentorial bleed (Table 3).

### Surgical Intervention

Among the 292 patients, 93 (31.8%) patients were subjected to surgery in which 54 patients (18.5%) underwent craniotomy and clot evacuation meanwhile 8 patients (2.7%) underwent craniectomy due to intra-operatively brain oedema. Of the eight patients that underwent craniectomy, four patients passed away and the remaining four patients had a poor MRS score. Moreover, among the 54 patients that underwent craniotomy, 31 patients had a good MRS outcome, 6 patients showed a poor MRS score while 17 patients passed away.

There were 52 patients (17.8%) who underwent EVD for IVH or HCP. Among these patients, 23 patients passed away and among the 39 patients that survived, only 24 patients had a good MRS score.

Patient that underwent EVD and craniotomy had a statistical significance for mortality and morbidity (Table 4).

### Outcomes

Among the 292 patients, 178 patients (61.0%) presented with a good outcome while 114 patients (39.0%) had a poor outcome. A total of 82 patients (29.1%) passed away at 30 days in this study (Table 5).

Using binary logistic regression analysis for the significant variable detected based on the result, gender (male versus female), ischaemic heart disease, chronic kidney disease with diabetes, location of the bleed in the brainstem, usage of concomitant aspirin and warfarin, and surgery were significant for mortality (Table 6).

## Discussion

The mean age of ICH was 48.9 years old based on the finding. The age distribution chart (Figure 1) shows that the highest incidence of ICH occurs between the age of 31 years old and 60 years old. The chart is skewed to the right since the mean is higher than the median. This study proves that the incidence of ICH in the patient (mean 48.9) is lower compared to the Malaysian Stroke Registry data that showed a mean age of 60.8 years old. Although the data in this study showed non-statistical significance for mortality, the impact of disability-adjusted life years (DALY) is unarguable as the younger Malaysian population is developing ICH in Malaysia. Age was of no significance in predicting mortality (*P* = 0.739) in this study, which was a similar finding from other studies (12, 13).

There were 73.6% Malay patients, 18.5% Chinese patients, 6.8% Indian patients and 1.1% others who presented with ICH. Kuala Lumpur population demographic census suggests that Malay ethnic are the majority population in Kuala Lumpur at 41.6% followed by Chinese at 39.1%, Indians at 9.2% and others at 10.1% (13). The slightly more male preponderance compared to the female by a ratio of 1.65:1 and the majority of Malay ethnicity can be explained by the demographic in Kuala Lumpur (13). Ethnicity (*P* = 0.247) and sex (*P* = 0.063) showed a non-statistical significant for mortality.

Although ICH patients had multiple risk factors, the presence of IHD has been statistically significant for mortality (*P* = 0.001). Binary logistic regression analysis demonstrated that IHD (OR = 0.007; *P* = 0.003) had a significant influence on mortality. In our study, only 8 patients among 30 patients with IHD survived. This suggests a systemic impact of chronic hypertension to the cardiac and renal system contributing to the general poorer outcome and mortality. A complication of hypertension such as pulmonary oedema and myocardial infarction contributes to the high rate of deaths among these patients.

Similar findings to predict mortality was noted in a patient with CKD and diabetes presenting with ICH (OR = 0.071; *P* = 0.031). In this study, only 5 patients out of 22 patients with CKD survived but 1 patient had a poor MRS outcome. The higher incidences of ICH in patients undergoing dialysis are undeniable since high urea tends to present with poor platelet aggregation. Moreover, brain perfusion related injury (hypoperfusion) occurs frequently in patients with CKD. Similar findings were reported in the choices for healthy outcomes in caring for ESRD (CHOICE) cohort in which fatality case in ICH patients with CKD was up to 90% (14).

Hypertension was consistently seen in most of the patients (99.0%). Intracranial bleed (ICB) occurs mainly because patients tend to default their anti-hypertensive treatment (15). The other possible reasons could be that acute symptoms of stroke occur without any warning sign compared to heart disease whereby subtle symptoms and signs may be noticed in patients during regular clinic follow up (16).

Our clinical observation that patients with anti-coagulant tend to fare poorer had mixed results in this study. Anti-coagulant showed no contribution to poor outcome or mortality (*P* = 0.064). However, the combination use of aspirin with warfarin contributes to poorer mortality (*P* = 0.007). A conclusion is unable to be made due to only two patients consumed both drugs hence skewing the results to be significant.

It is interesting to note that some variables that are significant in the univariate analysis do not achieve significance in the binary logistic regression. Severe GCS, the presence of midline shift, clot size more than 50 cc and the presence of hydrocephalus were among the variables.

Patient presenting with brainstem bleed had a strong predictive factor of mortality (OR = 0.001; *P* = 0.001). Only 4 out of 53 patients with brainstem bleed survived but these patients had a poor MRS outcome. This is because the location is inoperable and could present high morbidity due to the close proximity to vital structures. Patients with brainstem bleed were activity of daily living (ADL) dependent post-ICH and were predisposed to hospital-acquired infection thus, contributing to the poor mortality.

The presence of a clot size of more than 50 cc showed to be significant for mortality (*P* = 0.012) in the univariate analysis. However, further analysis using binary linear regression analysis showed that this parameter was not predictive for mortality. There is a possibility that patients with lobar bleed tend to present with bigger clot sizes and generally tend to have a better outcome. Further sub-analysis is needed to segregate patients into lobar and non-lobar bleed to predict the outcome for mortality more accurately. The reason why a patient with clot size more than 50 cc tend to fare poor provided lobar bleed has been excluded will be related to oedema and chronic compression on the vital structure. This resulted in poor GCS and ADL dependence of the patient.

A similar outcome was noted for the presence of hydrocephalus based on the CT brain (*P* = 0.004) to predict mortality in the univariate analysis whereas it was not predictive of mortality upon binary logistic regression (OR = 3.57; *P* = 0.187).

The presence of ICH also was noted to contribute towards mortality (OR = 0.198; *P* = 0.010). This congruent finding was noted as EVD insertion is mostly performed with the presence of IVH, thus contributing to a similar outcome to the surgical EVD group (OR = 0.010; *P* = 0.001) as shown in this study.

Among the surgical group, craniotomy, craniectomy and EVD insertion were risk factors for mortality (*P* = 0.001). A more detailed analysis with bigger sample size is needed to confirm the finding that surgery would be an indicator of poorer outcome. However, it is difficult to compare this finding to normal patients that have not undergone surgery. It is also possible that many of these patients that underwent surgery had poor pre-morbid with GCS drop. Therefore, further sub-analysis is needed to be done.

This study shows a 29.1% mortality rate and this is consistent with the Malaysian Stroke Registry mortality rate of 25.9% (2). This shows that although younger patients are common in presenting with ICH, the mortality rate is similar.

### Limitation

There are several limitations to this study. Firstly, the number is small and may not be statistically significant to study variables such as the effect of surgery on the prognosis. Secondly, a month duration to perform this study is not optimal to observe neurological recovery. It is also noticed that some patients with good GCS and borderline size clots tend to deteriorate later in day 3–day 5 due to oedema requiring surgery. Most GCS scoring for these patients was taken during admission, which may have obscured the result when the patient requires surgery. Moreover, GCS scoring is non-ideal for stroke patients. The use of the NIH stroke score (NIHSS) score may be superior since there are more variables. However, most of the local neurosurgery centre in Malaysia uses GCS as a referral index tool. Based on this study, comorbid plays a role in the prognosis of a patient with ICH. A more specific scoring system for ICH may be needed that include these risk factors for a more accurate prognosis.

## Conclusion

This study has performed the largest and latest study involving neurosurgical intervention among the ICH patients in Malaysia. The mortality rate of 29.1% at 30 days is acceptable with the most common risk factor is hypertension (99.0%). The most significant predictors of mortality are IHD and CKD. Furthermore, the 48.9 years old mean age as reported in this study should be taken into consideration for more aggressive steps in curbing this scenario. Treatment for ICH in Malaysia could be carried out by treating hypertension at all primary care centres and creating more awareness of the disease. This is the evidence showing that younger patients in Malaysia present with ICH resulting in the loss of the young workforce hence this should be addressed more seriously. Moreover, the risk factors mentioned in this study could be used to aid in the decision making by the clinicians to determine the outcome of ICH. The need for a more refine scoring system to include the comorbid and other parameters may be of help in future studies.

## Figures and Tables

**Table 1:** Demographic and clinical profile of patients with outcome based on MRS outcome and mortality (*n* = 292)

		Total patient		MRS outcome				Mortality			
	
				Good	Poor	*P*-value	Odd ratio	Alive	Death	*P*-value	Odd ratio

				MRS 1–3	MRS 4–6						
		
		*n*	%	*n*	*n*			*n*	*n*		
Age		*49.81±12.89		178	114	0.371	0.992	207	85	0.739	0.997
Gender											
	Male versus Female	182	62.3	105	77	0.142	1.447	122	60	0.063	0.598
	Female	110	37.7	73	37			85	25		
Race						0.949				0.247	
	Malay	215	73.6	132	83			155	60		
	Chinese versus Malay	54	18.5	32	22	0.774	1.093	39	15	0.985	1.006
	Indian versus Malay	20	6.8	11	9	0.576	1.301	13	10	0.055	0.387
	Others versus Malay	3	1.1	3	0	0.999	0.901	3	0	0.999	0.625
Hypertension		289	99.0	176	113			205	84		
	Systolic	*207.4±24.6		*208.0±24.8	*206.5±24.3	0.609	0.998	*207.2±24.7	*207.9±24.5	0.808	0.999
	Diastolic	*105.8±10.6		*105.4±10.7	*106.2±10.4	0.527	1.007	*105.2±10.8	*107.1±9.8	0.172	0.983
Risk factor						**0.001**				**0.001**	
	Diabetes (DM) versus None	78	26.7	50	28	0.282	1.370	61	17	0.713	0.883
	IHD versus None	10	3.4	2	8	**0.005**	9.787	2	8	**0.001**	0.062
	CKD versus None	8	2.7	1	7	**0.009**	17.128	1	7	**0.002**	0.035
	Valvular heart disease versus None	7	2.4	2	5	**0.034**	6.117	4	3	0.158	0.328
	DM+IHD versus None	14	4.8	5	9	**0.011**	4.404	5	9	**0.001**	0.137
	DM+IHD+CKD versus None	3	1.0	1	2	**0.199**	4.894	1	2	0.091	0.123
	DM+CKD versus None	8	2.7	2	6	**0.017**	7.340	3	5	**0.011**	0.148
	CKD+IHD versus None	1	0.3	0	1	1.000	3.95 × 109	0	1	1.000	0.000
	All versus None	1	0.3	0	1	1.000	3.95 × 109	0	1	1.000	0.000
	None	162	55.5	115	47			130	32		
Medication						0.321				0.161	
	Aspirin and warfarin versus None	2	0.7	1	1	0.689	1.765	1	1	0.460	0.349
	Warfarin versus None	15	5.1	7	8	0.190	2.018	10	5	0.528	0.699
	Aspirin versus None	51	17.5	27	24	0.150	1.569	30	21	**0.031**	0.499
	None	224	76.7	143	81			166	58		
Number of symptoms											
	LOC	193	66.1	107	86	0.837	1.075	128	65	0.886	1.057
	Headache	167	57.2	121	46	0.134	2.452	134	33	0.069	0.192
	Vomit	147	50.3	106	41	0.934	1.043	116	31	0.555	1.432
	Weakness	143	49.0	105	38	0.320	1.805	114	29	0.862	0.883
	Slurring of speech	125	42.8	90	35	0.472	0.691	98	27	0.606	1.648
	Seizure	2	70.0	0	2	0.999	0.001	2	0	0.999	0.001
GCS						**0.001**				**0.001**	
	Full	59	20.2	52	7			54	5		
	Mild versus Full	69	23.6	56	13	0.282	1.724	61	8	0.562	0.706
	Moderate versus Full	97	33.2	64	33	**0.003**	3.830	78	19	0.070	0.380
	Severe versus Full	67	22.9	6	61	**0.001**	75.52	14	53	**0.001**	0.024

Notes: statistical significance *P* < 0.05 shown in bold;

* mean ± standard deviation;

LOC = loss of consciousness

**Table 2:** Radiological imaging and the clinical significance on outcome based on MRS outcome and mortality (*n* = 292)

		Total patient		MRS outcome				Mortality			
	
				Good	Poor	*P*-value	Odd ratio	Alive	Death	*P*-value	Odd ratio

				MRS 1–3	MRS 4–6						
		
		*n*	%	*n*	*n*			*n*	*n*	*n*	
Location						**0.001**				**0.001**	
	Putamen versus Lobar	134	45.9	108	26	0.874	0.923	122	12	0.431	1.627
	Thalamic versus Lobar	45	15.4	21	24	**0.007**	4.381	31	14	0.098	0.354
	Cerebellar versus Lobar	31	10.6	26	5	0.649	0.737	25	6	0.565	0.667
	Brainstem versus Lobar	53	18.2	0	53	0.997	6.19 × 109	4	49	**0.001**	0.013
	Lobar	29	9.9	23	6			25	4		
Side						**0.001**				**0.001**	
	Right versus Other	125	42.8	98	27	**0.001**	0.116	110	15	**0.001**	8.75
	Left versus Other	86	29.5	56	30	**0.001**	0.226	70	16	**0.001**	14.667
	Other	81	27.7	24	57			27	54		
MLS											
	Less than 5 mm versus more than 5 mm	275	94.2	174	101	**0.003**	0.179	197	78	0.264	1.768
	More than 5 mm	17	5.8	4	13			10	7		
Clot size						**0.012**				**0.171**	
	Less than 15 cc versus more than 50 cc	142	48.6	86	56	0.212	0.434	95	47	0.656	1.348
	15 cc to 30 cc versus more than 50 cc	111	38.0	77	34	0.071	0.294	87	24	0.198	2.417
	30 cc to 50 cc versus more than 50 cc	29	9.9	11	18	0.908	1.091	19	10	0.754	1.267
	More than 50 cc	10	3.4	4	6			6	4		
IVH											
	Yes versus No	149	51.0	85	64	0.162	0.714	104	45	0.675	0.898
	No	143	49.0	93	50			103	40		
HCP											
	Yes versus No	55	18.8	24	32	**0.004**	2.397	30	25	**0.004**	0.407
	No	237	81.2	154	83			177	60		

Note: statistical significance *P* < 0.05 shown in bold; variable was tested using simple logistic regression

**Table 3:** CT imaging based on Kuroda & Kanaya Classification

			Total patient	

			*n*	%
Basal ganglia bleed	I	External capsule	11	6.1
	II	Capsular	18	10.1
	IIIa	Cp without V	13	7.3
	IIIb	Cp with V	42	23.5
	IVa	Ca + p without V	11	6.1
	IVb	Ca + p with V	39	21.8
	V	Thamalus or subthalamus	45	25.1

Thalamic bleed	Ia	Thalamus	5	
	Ib	Thalamus + V	18	
	2a	Internal capsule without V	5	
	2b	Internal capsule with V	13	
	IIIa	Hypothalamus/midbrain without V	2	
	IIIb	Hypothalamus/midbrain with V	2	

Note: V = ventricle; p = putamen; Ca = caudate; Cp = capsular

**Table 4:** Surgical intervention performed and clinical significance on outcome based on MRS outcome and mortality (*n*=292)

	Total patient		MRS Outcome				Mortality			
	
			Good	Poor	*P*-value	Odd ratio	Alive	Death	*P*-value	Odd ratio

			MRS 1–3	MRS 4–6						
		
	*n*	%	*n*	*n*			*n*	*n*	*n*	
Surgery	93	31.8	39	54	**0.001**	**3.208**	52	41	**0.001**	0.360
Type of surgery					0.001				0.001	
Craniotomy versus None	32	11.0	15	17	**0.016**	2.541	19	13	**0.035**	0.434
Craniectomy versus None	7	2.4	0	7	0.999	3.62 × 109	4	3	0.236	0.396
EVD versus None	28	9.6	7	21	**0.001**	6.726	10	18	**0.001**	0.165
Craniotomy + EVD versus None	22	7.5	16	6	0.730	0.841	18	4	0.617	1.335
VGSG + EVD versus None	1	0.3	1	0	1.000	0.000	1	0	1.000	4.79 × 109
Craniectomy + EVD versus None	1	0.3	0	1	1.000	3.62 × 109	0	1	1.000	0.000
None	201	68.8	139	62			155	46		

Notes: statistical significance *P* < 0.05 shown in bold; variable was tested using simple logistic regression

**Table 5:** MRS score at 30 days

	MRS	Total patient	

		*n*	%
Good	1 to 3	178	61.0
Poor	4 to 6	114	39.0

**Table 6:** Multivariant binary logistic regression analysis used to predict of ICH mortality and 95% CI relative risk of ICH

Variable	*P*-value	Adjusted OR (95% CI)	
**Gender (Male versus Female)**	**0.043**	**0.343**	**(0.122–0.968)**
**Risk factor**	0.072	0.066	(0.014 0.321)
Diabetes (DM) versus None	0.237	0.446	(0.117 1.702)
IHD versus None	**0.003**	0.007	(0.000 0.195)
CKD versus None	0.072	0.036	(0.001 1.344)
Valvular heart disease versus None	0.568	0.394	(0.016 9.658)
DM + IHD versus None	**0.002**	0.016	(0.001 0.222)
DM + IHD + CKD versus None	0.026	0.005	(0.000 0.532)
DM + CKD versus None	**0.031**	0.071	(0.006 0.789)
CKD + IHD versus None	1.000	0.001	(0.000)
All versus None	1.000	0.001	(0.000)
**Medication**	0.064		
Aspirin versus None	0.962	1.692	(0.000 3.52 × 10^9^)
Warfarin versus None	1.000	1.000	(0.082 12.252)
Aspirin and Warfarin versus None	0.007	14.696	(2.083 103.660)
**Location**	**0.001**		
Putamen versus Lobar	0.055	6.575	(0.964 44.865)
Thalamic versus Lobar	0.707	1.590	(0.141 17.873)
Cerebellar versus Lobar	0.393	0.266	(0.013 5.547)
Brainstem versus Lobar	0.001	0.001	(0.000 0.013)
**IVH: Yes versus No**	**0.049**	0.198	(0.039 0.991)
**Type of surgery**	**0.001**		
Craniotomy versus None	**0.001**	0.017	(0.003 0.108)
Craniectomy versus None	**0.001**	0.010	(0.001 0.116)
EVD versus None	**0.001**	0.010	(0.001 0.135)
Craniotomy + EVD versus None	0.359	0.250	(0.013 4.833)
VGSG + EVD versus None	1.000	1.16 × 107	(0.000)
Craniectomy + EVD versus None	0.999	0.000	(0.000)
